# Perioperative infusion of low- dose of vasopressin for prevention and management of vasodilatory vasoplegic syndrome in patients undergoing coronary artery bypass grafting-A double-blind randomized study

**DOI:** 10.1186/1749-8090-5-17

**Published:** 2010-03-28

**Authors:** Georgios Papadopoulos, Eleni Sintou, Stavros Siminelakis, Efstratios Koletsis, Nikolaos G Baikoussis, Efstratios Apostolakis

**Affiliations:** 1 Department of Anesthesia and Intensive Care Unit, University Hospital of Ioannina, Ioannina, Greece; 2 Cardiac Surgery Department, University Hospital of Ioannina, Ioannina, Greece; 3 Department of Cardiothoracic Surgery Department, Patras University Hospital Patras, Greece

## Abstract

Preoperative medication by inhibitors of angiotensin-converting enzyme (ACE) in coronary artery patients predisposes to vasoplegic shock early after coronary artery bypass grafting. Although in the majority of the cases this shock is mild, in some of them it appears as a situation, "intractable" to high-catecholamine dose medication. In this study we examined the possible role of prophylactic infusion of low-dose vasopressin, during and for the four hours post-bypass after cardiopulmonary bypass, in an effort to prevent this syndrome. In addition, we studied the influence of infused vasopressin on the hemodynamics of the patients, as well as on the postoperative urine-output and blood-loss. In our study 50 patients undergoing coronary artery bypass grafting were included in a blind-randomized basis. Two main criteria were used for the eligibility of patients for coronary artery bypass grafting: ejection fraction between 30-40%, and patients receiving ACE inhibitors, at least for four weeks preoperatively. The patients were randomly divided in two groups, the group A who were infused with 0.03 IU/min vasopressin and the group B who were infused with normal saline intraoperativelly and for the 4 postoperative hours. Measurements of mean artery pressure (MAP), central venous pressure (CVP), systemic vascular resistance (SVR), ejection fracture (EF), heart rate (HR), mean pulmonary artery pressure (MPAP), cardiac index (CI) and pulmonary vascular resistance (PVR) were performed before, during, and after the operation. The requirements of catecholamine support, the urine-output, the blood-loss, and the requirements in blood, plasma and platelets for the first 24 hours were included in the data collected. The incidence of vasodilatory shock was significantly lower (8% vs 20%) in group A and B respectively (p = 0,042). Generally, the mortality was 12%, exclusively deriving from group B. Postoperatively, significant higher values of MAP, CVP, SVR and EF were recorded in the patients of group A, compared to those of group B. In group A norepinephrine was necessary in fewer patients (p = 0.002) and with a lower mean dose (p = 0.0001), additive infusion of epinephrine was needed in fewer patients (p = 0.001), while both were infused for a significant shorter infusion-period (p = 0.0001). Vasopressin administration (for group A) was associated with a higher 24 hour diuresis) (0.0001).

In conclusion, low-dose of infused vasopressin during cardiopulmonary bypass and for the next 4 hours is beneficial for its postoperative hemodynamic profile, reduces the doses of requirements of catecholamines and contributes to prevention of the postcardiotomy vasoplegic shock in the patient with low ejection fraction who is receiving ACE preoperatively.

## Background

Coronary artery bypass grafting by using cardiopulmonary bypass (CPB) may be complicated by persistent hypotension due to low systemic vascular resistance, in 5-22% of patients [1,2]. Different causes have been associated with this situation, like hypothermia and duration of CPB, total cardioplegic volume infused, reduced left ventricular function, preoperative treatment with angiotensin-converting enzyme inhibitors, and systemic inflammatory response syndrome (SIRS), or inappropriate low arginine-vasopressin secretion. On the other hand, different factors such as the reduced effect on the pressor catecholamines, cellular acidosis, opening of ATP sensitive channels, efflux of K+ and hyperpolarization of the myocytes, which prevents Ca++ channels from opening [3,4].

An advanced form of this post-cardiotomy hypotension is the so-called vasodilatory or vasoplegic shock which is a life-threatening condition, intractable in the usual management with fluid administration, inotropes, and even vasopressor catecholamines [4-7]. The incidence of this syndrome is reported to range between 8.8 to 10% [8-10], but in patients with preoperative severe left ventricular systolic dysfunction it may be observed up to 42% of the cases [11]. In addition, the infusion of catecholamines often complicates the cardiovascular stabilization by producing arrhythmias and entering into a circulus vicious [12,13].

Vasopressin has been introduced as adjunctive to catecholamines in cardiac arrest and in advanced vasodilatory shock, and the results have shown that it is more effective than vasopressor catecholamines [6,13,14].

We examined the effectiveness of intraoperative infusion of arginine vasopressin in operated cardiac patients to prevent the postoperative vasodilatory chock. The aim of our study was to investigate the effects of prophylactic administration of low-dose of vasopressin (of 0.03 Units per minute for 4 hours), on the patients' hemodynamic status, on the incidence of vasodilatory shock, and on urine output and blood loss, for the 1^st ^day after the operation.

## Materials and methods

This study was conducted following approval from the Ethics Committee and our hospital's Scientific Committee and after having obtained written informed consent from all patients. A total of 50 patients, aged 32 to 81 years (61 ± 16 years), were operated between January 2003 to December 2005 for coronary artery disease. All the patients underwent selective coronary artery bypass grafting by the same anesthetic and surgical team. The inclusion criteria for the patients were the following:

1. Patients were on ACE inhibitors therapy for at least 4 weeks prior to surgical procedure, and

2. Patients had impaired left ventricular ejection fraction, expressed by a preoperatively estimated injection fraction between 30-40% (by transthoracic or transesophageal echo).

From the study patients were excluded, according to the following criteria:

1. injection fraction less than 30%,

2. in shock or critical hemodynamic state, confirmed by the introduced TEE. In addition, patients with appearance of shock or severe hemodynamic instability "intractable" in simple preload-manipulations (fluids infusion) and in combination with simultaneous (observed by TEE) impairment of left ventricular function during the operation and in the first 2 hours after termination of cardiopulmonary bypass, were excluded,

3. confirmed hepatic, and/or renal, and/or thyroid, and/or adrenal disease,

4. significant carotid stenosis or any event of intraoperative brain ischemia documented by continuous transcranial SvO_2 _(INVUS),

5. significant peripheral obstructive arteriopathy,

6. documented pulmonary hypertension, expressed by systolic pulmonary pressure >30-35 mm Hg, and

7. chronic obstructive pulmonary disease, confirmed by preoperative spirometry, thorax X-rays and blood gas analysis.

For all patients a double right internal jugular vein catheterization was performed, with placement of a three-way central catheter, as well as a Swan - Ganz catheter for continuous measurement of pulmonary artery pressure, cardiac output and mixed venous blood saturation. Next, a urinary catheter was introduced for measurement of hourly diuresis. In addition, a transesophageal ultrasound probe was introduced for intra- and post-operative estimation of cardiac function. All three catheters were retained for the first 24 h and removed in ICU after this time.

Induction of anesthesia was performed using a continuous remifentanyl infusion at a dosage of 0.5 μg/Kg/min, intravenous etomidate at a titrated dosage of 0.2-0.3 mg/Kg, and 0.6 mg/kg of rocuronium. For maintenance of anesthesia, the following were used: remifentanyl, at a dose of 0.25-0.5 μg/Kg/min, sevoflurane, 1-2%, and rocuronium in continuous infusion at a rate of 20 mg/h. The operation was performed using cardiopulmonary bypass, systemic hypothermia at 30°C, and intermittent (after each distal anastomosis) application of cold blood cardioplegia in the same manner. Patients were divided in a blind- manner in two groups. In group A, continuous infusion of a solution of vasopressin (Pitressin, Pfizer, Kalsruhe, Germany) 0.03 IU/min was intravenously administered through a central line at an infusion rate of 22 ml/h. The infusion began 20 minutes before beginning cardiopulmonary bypass and was continued throughout the operation for the next 4 hours after termination of the cardiopulmonary bypass. In group B, a solution of normal saline was administered in the same dose, way, and duration. Both solutions were prepared by a nurse, and infused at an infusion rate of 22 ml/h. Neither the surgeon nor the anesthetist or any other in the operating room except from this nurse did know the kind of infused solution, in each patient.

Ten minutes before termination of the cardiopulmonary bypass, a solution of norepinephrine, at a dose of 0.03 μg/Kg/min was routinely administered (in continuous iv infusion), and it was individually increased up to 0.05 μg/Kg/min during the next 24 hours until extubation, depending on the hemodynamic state of each patient. An additional dose of epinephrine of 0.01-0.03 μg/Kg/min was selectively infused in patients to whom the above dose of norepinephrine was insufficient in order to restore a normal cardiac output, whereas in every patient with vasodilatory shock.

After successful termination of the cardiopulmonary bypass and the followed homeostasis, the patients were transferred to the ICU, where the vasopressin or saline solution was continued, until completion of the pre-specified infusion-time (4 hours after termination of cardiopulmonary bypass). All the patients were sedated for the first 12-18 hours, and then they were extubated in the absence of any hemodynamic instability. For maintenance of sedation, a solution of Propofol in a dose of about 40 mg/h was continuously administered until the time of extubation. Postoperative urine output and blood loss from drains were hourly recorded, for the first 24 hours.

In all patients, the hemodynamic profile was routinely recorded, at five phases. The first phase (phase-1) was recorded at 20 minutes prior to initiation of extracorporeal circulation. The second (phase-2) was recorded at 20 minutes after termination of the cardiopulmonary bypass. The third phase (phase-3) was recorded at 40 minutes following termination of the cardiopulmonary bypass. The fourth phase (phase-4) was recorded at 60 minutes after termination of the cardiopulmonary bypass. Finally, the last phase (phase-5) was recorded at 2 hours following transfer of the patient in ICU. The recorded parameters of hemodynamic profile were the following: EF, HR, MAP, MPAP, CO, CVP, SVR, and PVR. The rest of the data which were recorded and were considered for the analysis of the results were the following:

1. The preoperative medication,

2. Biometric data such as age and BSA,

3. Some intraoperative factors such as cardiopulmonary bypass-time and ischemia-time,

4. The units of administered blood and/or blood products,

5. The 24-hour patient dieresis,

6. The 24-hour blood-loss, and

7. Requirement for inotropes and their dosage, as well as the mean dose and duration of norepinephrine administration.

### Statistical analysis

All data are expressed as mean value ± standard deviation. Values in both groups passed the Kolmogorov-Smirnof test for normality. Comparisons of continuous variables between groups were performed using the unpaired student's t-test. Comparison of categorical data between the two groups of patients was performed by the chi-square test or the Fischer's exact test, where appropriate. p-values less than 0.05 were considered statistically significant. All analyses were performed using the SPSS 16 statistical package.

## Results

Three patients died (6%) in the postoperative period (48 hours, 88 hours and 4 days postoperatively), all of them from the group B (12%) (0% versus 12%, p = 0.235). The cause of death for all patients was the multiple organ-system failure.

At first, the comparison between two groups was made regarding the general characteristics (sex, mean age, and BSA), clinical preoperative data (co-morbidity, severity of CAD and intraoperative hemodynamic measurements), preoperative medication and intraoperative data (cardiopulmonary bypass-time, ischemia-time, grafts number per patients, etc). All these data are presented in table 1 and 2. In table 3 the postoperative data (mortality, hemodynamic profile, needed inotropic support, etc) for the two groups is shown.

**Table 1:** The comparative pre- operative data from the patients both groups.

	***Group A***	***Group B***	***p***
***General characteristics***			
**Number of patients**	25	25	-
**Male/female**	20/5	21/4	1
**Age (y/s)**	66 ± 13	62 ± 15	0,319
**Height (cm)**	164 ± 9	168 ± 11	0,166
**Weight (kg)**	75 ± 11	72 ± 8	0,276
**BSA**	1.74 ± 7.4	1.82 ± 6.6	0,968
***Clinical preoperative data***			
**Hypertension**	16	14	0.773
**Diabetes mellitus**	8	10	0.769
**Euroscore**	4.8 ± 2.2	4.5 ± 2.6	0,662
**3-coronary vessel disease**	19	17	0.754
**2-coronary vessel disease**	6	9	0.538
**Significant Left main CAD**	2	4	0.667
**Ischemic mitral regurgitation 1+/4+**	7	4	0.496
**Ischemic mitral regurgitation 2+/4+**	4	8	0.321
**Ejection fraction 30-35%**	9	12	0.567
**Ejection fraction 35-40%**	16	13	0.567
**Cardiac Index (L/min/m^2^)**	3.1 ± 0,6	3.2 ± 0.8	0,619
**PCWP < 15**	9	10	0.773
**PCWP > 15**	16	15	0.773
***Preoperative medication***			
**aMEA**	25	25	-
**b-blockers**	14	17	0.377
**Calcium channel blockers (pts)**	11	8	0.561

**Table 2:** The comparative intra-operative data from the patients both groups.

	***Group A***	***Group B***	***P***
**Total vasopressin infused (U)**	12.4 ± 1.3	-	-
**Vasopressin's infusion-time (min)**	404 ± 33	-	-
**Operation's-time (min)**	238 ± 32	228 ± 26	0,231
**Cardiopulmonary bypass-time (min)**	169 ± 29	177 ± 20	0,262
**Myocardial ischemia-time (min)**	52 ± 14	47 ± 12	0.182
**Mean hypothermia (°C)**	31.4 ± 1.8	31.1 ± 1.5	0,525
**LIMA-used (pts)**	23	24	1.000
**Radial artery used (pts)**	9	6	0.538
**3-grafts bypass**	16	18	0.762
**2-grafts bypass**	9	6	0.538
**1-graft bypass (LIMA)**	-	1	1.0

**Table 3:** The post-operative data from the patients both groups

***Characteristics***	***Group A***	***Group B***	***p***
***Mortality***			
**Surgical mortality**	0(%)	3 (12%)	0.235
***Hemodynamic profil***			
**Cardiac Index (L/min/m^2^)**	3.2 ± 0.7	3.0 ± 0.8	0,352
**Heart rate (/min)**	78 ± 11	83 ± 9	0,085
**PVR**	198 ± 40	214 ± 29	0,112
**Mean PAP (mm Hg)**	21 ± 4	19 ± 4	0,084
**Mean AP (mm Hg)**	84 ± 11	78 ± 7	**0,026**
**SVR (dyn.cm/m^2^)**	1210 ± 102	1103 ± 123	**0.002**
**CVP (mm Hg)**	8.5 ± 2.5	7 ± 1.8	**0.019**
**EF**	38.0 ± 3.9	35.5 ± 4.1	**0.032**
**Vasodilatory shock (pts)**	1 (8%)	6 (20%)	**0.042**
***Inotropic needs***			
**Needed norepinephrine (pts)**	6	18	**0.002**
**Needed additional epinephrine (pts)**	5	17	**0.001**
**Mean catecholamine infusion-time (Hours)**	10 ± 4	18 ± 6	**0,000**
**Mean norepinephrine-dose μg/Kg/min**	0.16 ± 0.04	0.44 ± 0.07	**0,000**
***Blood-loss and urine output***			
**Mean blood loss (ml)**	650 ± 125	975 ± 100	**0,000**
**Mean urine volume (ml)**	5603 ± 1450	3910 ± 1102	**0.000**
***Transfusion needs***			
**Mean erythrocytes' units transfused**	3.1 ± 1.7	4.2 ± 1.8	**0.031**
**Mean plasma's units transfused**	6.1 ± 2.3	5.8 ± 3.1	0.699
**Mean platelets' units transfused**	4.3 ± 1.8	5.7 ± 2.1	**0,015**

**Figure 1:**
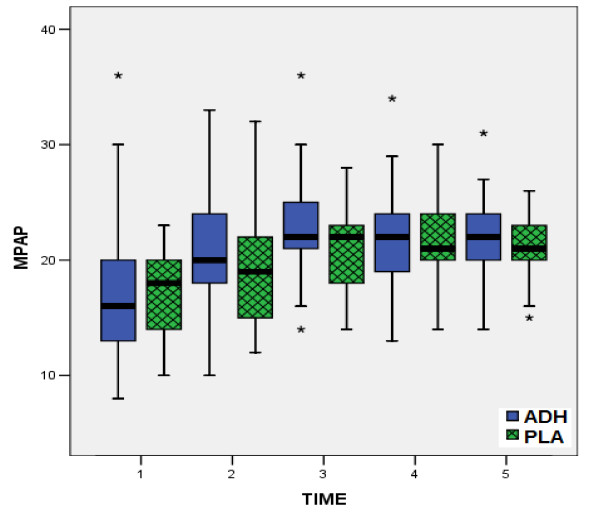
**Mean Pulmonary Pressure during time-points T1 - T5**. Distribution of values for mean pulmonary pressure (MPAP) during time-points T1 - T5 for group I (vasopressin, in blue boxplots) and group II (placebo, in green boxplots). (median = black line, boxplot = 50% of data set, lines on both sides of the boxplot = dispersion for 99% of values, * = numbers outside of distribution range for 99% of values).

**Figure 2:**
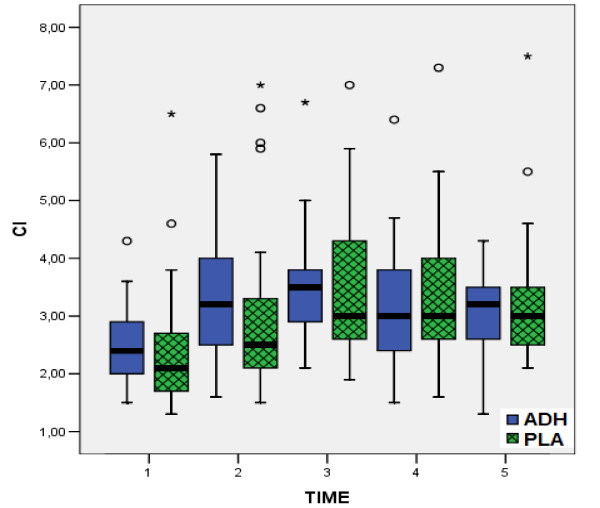
**Cardiac Index during time-points T1 - T5**. Distribution of values for cardiac index (CI) during time-points T1 - T5 for group I (vasopressin, in blue boxplots) and group II (placebo, in green boxplots). (median = black line, boxplot = 50% of data set, lines on both sides of the boxplot = dispersion for 99% of values, * = numbers outside of distribution range for 99% of values).

**Figure 3:**
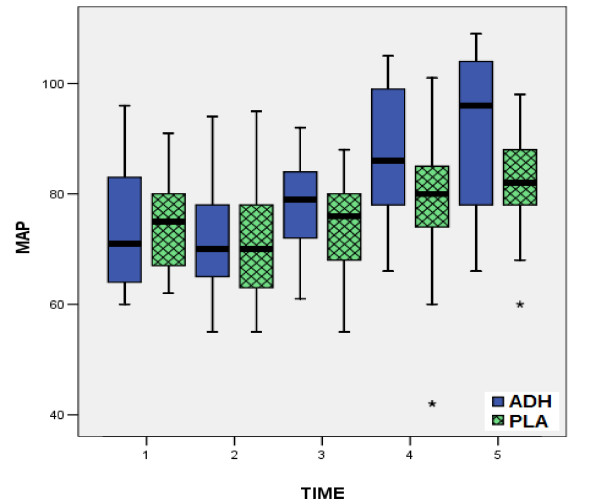
**Mean arterial pressure values during time-points T1 - T5**. Distribution of mean arterial pressure (MAP) values during time-points T1 - T5 for group I (vasopressin, in blue boxplots) and group II (placebo, in green boxplots). (median = black line, boxplot = 50% of data set, lines on both sides of the boxplot = dispersion for 99% of values, * = numbers outside of distribution range for 99% of values).

**Figure 4:**
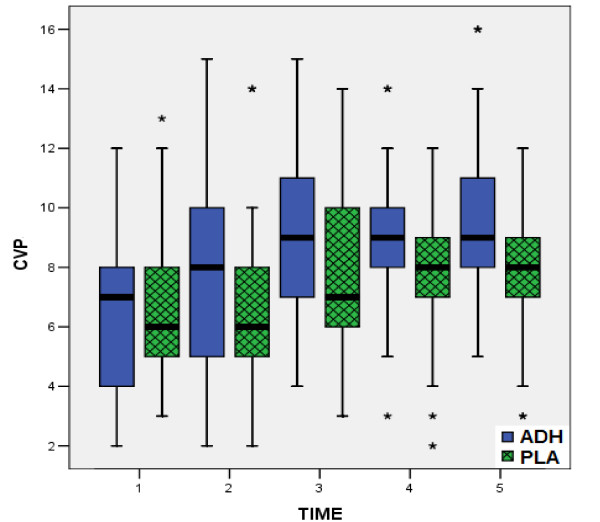
**Central Venous Pressure during time- points T1 - T5**. Distribution of values for central venous pressure (CVP) during time- points T1 - T5 for group I (vasopressin, in blue boxplots) and group II (placebo, in green boxplots). (median = black line, boxplot = 50% of data set, lines on both sides of the boxplot = dispersion for 99% of values, * = numbers outside of distribution range for 99% of values).

**Figure 5:**
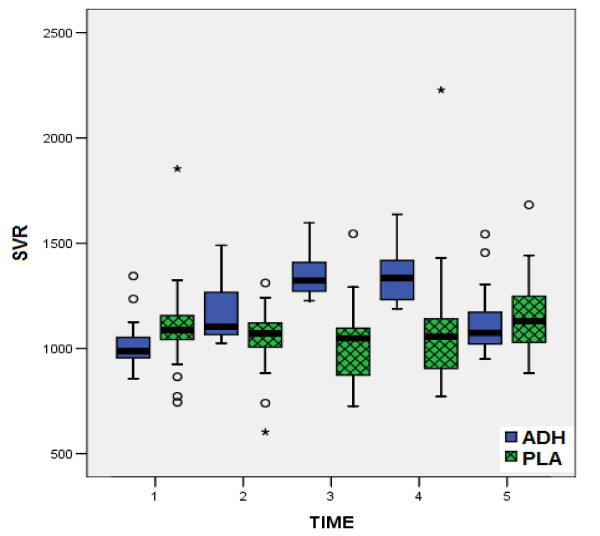
**Systemic Vascular Resistance during time-points T1 - T5**. Distribution of values for peripheral resistance (SVR) during time-points T1 - T5 for group I (vasopressin, in blue boxplots) and group II (placebo, in green boxplots). (median = black line, boxplot = 50% of data set, lines on both sides of the boxplot = dispersion for 99% of values, * = numbers outside of distribution range for 99% of values).

**Figure 6:**
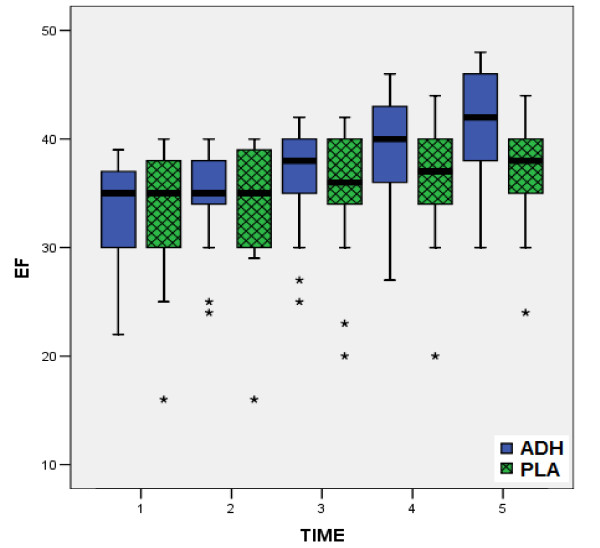
**Left ventricular Ejection Fraction during time-points T1 - T5**. Distribution of values for left ventricular ejection fraction (E.F.) during time-points T1 - T5 for group I (vasopressin, in blue boxplots) and group II (placebo, in green boxplots). (median = black line, boxplot = 50% of data set, lines on both sides of the boxplot = dispersion for 99% of values, * = numbers outside of distribution range for 99% of values).

According to all preoperative data, there were no statistically significant differences between the two groups, confirming the similarity of the groups at baseline (table 1). In the same way, from the comparison of postoperative measurements (table 3), no statistical significant differences were observed between two groups, concerning the factors HR, MPAP (fig. 1), CI (fig. 2) and PVR. On the contrary, comparison of values of MAP (fig. 3), CVP (fig. 4), SVR (fig. 5), and EF (fig. 6) following extracorporeal circulation showed significantly higher values in group A (table 2).

The mean vasopressin's infusion-time was 404 ± 33 minutes and the mean total dose of infused vasopressin in the patients of group A were 12.4 ± 1.3 Units (table 2).

Vasodilatory shock is considered the hemodynamic state characterized by a systolic arterial pressure of less than 80 mmHg (or mean arterial pressure < 70 mm Hg), despite of a cardiac output more than 5 L/min (or a cardiac index > 2.5 L/min/m^2^) (9, 10). According to this definition, one (1) patient of the vasopressin group (4%), and six (6) patients of the control group (24%) developed vasodilatory shock, during the first 24 hours of postoperative observation (p = 0.042) (table 3).

It is of note that in none of the patients a hypertensive crisis was observed. Inotropes infusion (norepinerhrine and/or epinephrine) was individually decided, depending on the postoperative hemodynamic status of the patients for the first 24 hours. Norepinephrine was infused in a minimal dose of 0.03-0.05 μg/Kg/min in 6 patients (24%) of group A and in 18 patients (72%) of group B (p = 0.002). Epinephrine infusion was additionally necessary in 5 patients (20%) of group A and in 17 (68%) of group B (p = 0.001). Generally, the catecholamine infusion-time was significantly lower in group A (10 ± 4 hours), in comparison to group B (18 ± 6 hours) (p = 0.0001) (table 3). Mean needed doses of norepinephrine were significantly lower in group A (0.16 ± 0.04 μg/Kg/min) than in group B (0.44 ± 0.07 μg/Kg/min) (p = 0.0001) (table 3).

Postoperative urine output during the first 24 hours was significantly higher in group A (5603 ± 1450 ml), in comparison to group B (3910 ± 1102 ml (p = 0.0001) (table 3).

The needed transfusions for blood and platelet units were statistically significantly lower for the patients of group A, in comparison to group B, in contrast to transfused plasma units. Moreover the postoperative blood loss for the first 24 hours was significantly lower in group A (650 ± 125 ml), compared to group B (975 ± 100 ml) (p = 0.0001) (table 3).

## Discussion

The vasodilatory shock is a state of abrupt hemodynamic deterioration in the first hours following open heart surgery. It is mainly characterized by a vasodilatory hypotension (systolic BP < 80 mmHg, while cardiac output is restored >5 L/min) associated with lactic acidosis, tachycardia, decreased systemic vascular resistance and low filling pressures [11,15,16]. The hypotension is characteristically unresponsive either to catecholamine administration (or necessitating norepinephrine administration more than 8 μg/min), or to preload increase by excessive fluid infusion [17].

This situation is attributed to a loss of vascular tone, due to either the inflammatory mediators produced by the cardiopulmonary bypass or the administered vasodilators such as phosphodiesterase inhibitors, nitrates, etc [5,16]. Some factors such as congestive heart failure (with EF < 35%), preoperative use of angiotensin-converting enzyme inhibitors and/or b-blockers and/or amiodarone and phosphodiesterase inhibitors, seem to be related with increased postoperative incidence of the vasodilatory shock [11,15,18-20]. In our study, the influence of low-dose of vasopressin on postoperative vasodilatory shock was examined in patients with two predisposing factors of this syndrome: low ejection fraction and preoperative administration of ACE inhibitors. In fact, according to Argengiano et al [11], both low ejection fraction and use of ACE inhibitors were independent risk factors for the development of postoperative vasodilatory shock. In fact, while the incidence of vasodilatory shock in patients with a normal ejection fraction was 3.3%, in patients with a low ejection fraction or receiving ACE inhibitors, it was 26.9% and 26.7%, respectively [11]. In our study, the incidence of vasodilatory shock was significantly lower in the group of vasopressin, being 20% in the control group and 4% in the vasopressin group (table 3), and much lower from those values reported by Argengiano et al [21]. According to this study, which included patients with end-stage heart failure who were subjected to left ventricular assist device placement, the incidence of postoperative vasodilatory shock was 42% [21].

The mortality of post-cardiotomy vasodilatory syndrome is high, dependent on its responsiveness in simultaneous vasopressin and norepinephrine infusion [7,22]. According to Gomes W, et al [8], the duration of norepinephrine refractory vasoplegia -it may persist for longer than 36-48 hours- significantly influences outcomes, because the syndrome may complicate postoperative oozing that requires blood and plasma transfusions. Generally, the mortality for post-cardiotomy patients may be increased up to 25% [8,9]. In our study, although the mortality for the patients of group A was 0% and for group B 12% this difference wasn't statistically significant. Of note, the mortality was not obviously related to the syndrome, all deaths occurred in patients with the syndrome, and at a later phase. Therefore, the calculated mortality for the patients suffering from the postcardiotomy vasoplegic shock syndrome was 50% (3 from the 6 pts) (table 3). The relative low mortality in our study may be attributed to the design of our protocol: we used a very-low dose of infusion; we started it 20 minutes before cardiopulmonary bypass in combination with norepinephrine infusion at the termination of cardiopulmonary bypass. Indeed, Patel B, et al [23] considers the low dose of 0.03 IU/min, in combination with its gradual starting of infusion as a factor of its effectiveness. In addition, another study has shown that the combined infusion of vasopressin with norepinephrine in post-cardiotomy patients did not cause an increase in mortality as predicted by Euroscore [24]. According to this study, the safety of low dose of vasopressin (≤0.04 IU/min) combined with norepinephrine was supported by the authors' observation that none of patients receiving vasopressin below 2 U/h (0.033 IU/min), died [24].

Concerning the appropriate dose of vasopressin there is not enough knowledge. It is mainly dependent on the indication, namely the management of postoperative vasodilatory shock or the prevention of the shock. For management, it has been used by several investigators in different dosages, between 2-6, or even 15 U/h [11,16,21]. Others have administered much lower dosages as these of 0.03-1 U/h [16,25-29]. However, infusion at a dose of about 6 U/hr seems to be effective, because it obtains a plasma level of ≥150 pg/ml and further increasing these levels does not offer additional benefit [11,16,17,25]. In fact, Mutlu G and Factor P [29], consider as appropriate the dose of <0.04 U/min and showed that it is safe and effective, even for the treatment of the septic vasodilatory shock. Higher dosages of vasopressin may be associated with several complications such as decreased coronary blood flow and cardiac output, ventricular arrhythmias and gut ischemia [28]. However, Torqersen C, et al [30] in their randomized and controlled trial by comparing two doses of 0.033 and 0.067 IU/min of arginine vasopressin infusion in patients with advanced vasodilatory shock, they showed that the patients receiving dose of 0.067 IU/min required significantly less norepinephrine, developed lower metabolic acidosis, without significant differences in MAP-levels, rate of adverse events and ICU-mortality, even for the 48 hours after the operation.

Our study showed, that intraoperative total "ultra-low" dose of 12.4 ± 1.3 Units of vasopressin may prevent the postoperative vasodilatory shock. Indeed, this "ultra-low" dose of vasopressin according to our study, obtains a significant increase of MAP (fig. 3), CVP (fig. 4), as well as a significant increase of SVR (fig. 5). The increased arterial pressure and systemic vascular resistance are mainly due to the produced by vasopressin systemic vasoconstrictive action, rather in patients in shock than in patients with a normal hemodynamic state [15,28]. Indeed, several studies in the past have shown that the perioperative administration of vasopressin restores the vascular tone in patients following cardiopulmonary bypass, especially in cases that are refractory to norepinephrine [16,21,26]. This result could be warranted by the known action of vasopressin: in low doses it has little or no influence on blood pressure of the normotensive patients, while the same doses in patients in vasodilatory shock produce an effective constrictive vessel action [15]. The increased cardiac index is attributed not only to the preload and after load changes [11,21,26,25,31], but also to the increased myocardial contractility. In fact, vasopressin infusion in advanced vasodilatory shock tends to improve myocardial performance by increasing of intramyocardial calcium concentrations, and producing coronary artery vasodilatation, in combination with the increase of myocardial blood flow due to increased systemic perfusion pressure [12,14]. The observation of significant postoperative increase of ejection fraction in our patients receiving vasopressin (fig. 6), is confirmed only by our findings, as to the best of our knowledge, no other study has recorded and evaluated this hemodynamic parameter.

Our study also showed that pulmonary vascular resistance and mean pulmonary artery pressure were not affected by the vasopressin infusion (fig. 1). It may attributed to the observed vasodilatory effect of vasopressin in the pulmonary vasculature [21,31], influence (of action) which is already experimentally confirmed and is due to a release of NO by the endothelial pulmonary capillaries [32]. Because of the above described action, vasopressin has been successfully used by Tayama E, et al [32], in cardiac surgical patients with preoperative pulmonary hypertension.

Concerning the postoperative needs of norepinephrine, our data showed that in the vasopressin group the percentage of patients requiring administration was significantly lower in comparison to the control group (6 pts or 24% versus 18 pts or 72%) (table 3). Similarly, an impressive difference was observed in the number of patients requiring additive infusion of epinephrine. While in the group of vasopressin only 5 pts (20%) required additional infusion of epinephrine, in the group of placebo it was 17 pts (68%) (table 3). An even more impressive observation was the difference to the mean administered dose of norepinephrine: this was significantly lower (0.16 ± 0.04 versus 0.44 ± 0.07 μg/min) in the vasopressin group. Similarly, the mean-time of catecholamine's infusion was significantly lower in the vasopressin group (10 ± 4 versus 18 ± 6 hours) (table 3).

Several studies have demonstrated the augmented vasoconstrictor action of vasopressin in patients with hypotension not responding to high-dose of norepinephrine, dopamine and fluid resuscitation [33], action which persists for up to 2 hours [34] and with a serious advantage: less pronounced vasoconstriction in the coronary and cerebral circulation [35]. Finally, to this double beneficial action of vasopressin for myocardium and brain, the protective action for the kidneys can be added. Experimental studies in protocols of short and prolonged cardiac arrest have shown that vasopressin produced a significantly higher vital organ blood flow and cerebral oxygen delivery than epinephrine did [36,37].

The increased urine output represents a remarkable result of infused vasopressin due -according to several studies- to the increased mean arterial pressure of the patient and therefore to the improvement of glomerular filtration rate [37,38]. However, Bragadottir G, et al [35] showed, that low to moderate doses of vasopressin (0.03-0.08 IU/min) in post-cardiac surgery patients cause a significant renal vasoconstricion and a decline in renal blood flow, which was accompanied by an increased glomerular filtration rate, suggesting that vasopressin mainly constricts efferent arterioles. It is of note that although these patients were not in shock, vasopressin infusion seems to produce an impairment of the renal oxygen demand/supply relationship [39]. Several studies have shown that vasopressin receptors in the renal vasculature are located in the efferent arterioles, in contrast to the catecholamine receptors, which are located in the adducing arterioles [33,35]. Therefore, although the vasoconstrictive action of catecholamines leads to a decrease in the filtration fraction, the action of vasopressin leads to an increase in the filtration fraction and, hence, to an increase of urine output [33,37,38].

We also observed this significantly increased 24-hour diuresis in the patients of the vasopressin group (table 3). Morales D, et al [16], proposed the long-term administration (up to 12 hours) of vasopressin in patients with postcardiotomy vasodilatory shock associated with renal insufficiency (instead that of 2 to 3 hours in patients with normal renal function), to maintain an improved filtration rate and urine output.

Although vasopressin causes a decrease of platelets in a significant number of patients (up to 52%) [12,40], it enhances blood coagulation. This can be attributed to an observed increase the plasma concentrations of factors VIII and von Willebrand [41,42]. The combination of several factors as those just mentioned, like the produced vasoconstriction and probably the increased adhesion of platelets (there are receptors V1 on them) [41], may explain the observed statistically significant reduced post-cardiotomy blood loss in the vasopressin group, in our study (table 3), this finding is in accordance to the less transfusions needs in the group A. Because desmopressin -a known drug already used for the reduction of postoperative bleeding in post-cardiotomy patients is an analogue of vasopressin [36], could potentially offer an additive "hemostatic role" in the vasopressin actions.

## Conclusions

In summary, infusion of an "ultra-low" dose vasopressin (0.03 U/min) during cardiopulmonary bypass and for the first four hours after coronary artery bypass grafting in patients with preoperative medication with ACE inhibitors who are having low ejection fraction, is safe and beneficial. It significantly reduces the required doses of catecholamines, obtaining a better hemodynamic profile, a higher urine output and lower blood loss for the first 24 hours. The use of an "ultra-low" dose vasopressin seems to be preventive for the incidence of observed post-cardiotomy vasodilatory shock. Finally, it may decrease both catecholamine dose and duration of their administration, it is considered as a useful agent for decreasing all their side-effects.

## Competing interests

The authors declare that they have no competing interests.

## Authors' contributions

All authors: 1) have made substantial contributions to conception and design, or acquisition of data, or analysis and interpretation of data; 2) have been involved in drafting the manuscript or revising it critically for important intellectual content; and 3) have given final approval of the version to be published.
